# *Vitreoscilla massiliensis* sp. nov., Isolated From the Stool of an Amazonian Patient

**DOI:** 10.1007/s00284-021-02577-8

**Published:** 2021-06-24

**Authors:** Sokhna Ndongo, Mossaab Maaloum, Magali Richez, Rachid Saile, Pierre-Edouard Fournier, Jean Christophe Lagier, Didier Raoult, Saber Khelaifia

**Affiliations:** 1 IHU-Méditerranée Infection, Marseille, France; 2 IRD, MEPHI, APHM, Institut Hospitalo-Universitaire Méditerranée Infection, Aix Marseille Univ, 19-21 Boulevard Jean Moulin, 13385 Marseille Cedex 05, France; 3 IRD, AP-HM, SSA, VITROME, IHU-Méditerranée Infection, Aix Marseille Univ, Marseille, France; 4 Laboratory of Biology and Health, Faculty of Sciences Ben M’sik, Hassan II University, Casablanca, Morocco

## Abstract

Strain SN6^T^ is a non-motile and non-spore-forming gram-negative bacterium which was isolated from the stool sample of an Amazonian patient. The optimum growth was observed at 37 °C, pH 7, and 0–5 g/l of NaCl. Based on the 16S rRNA gene sequence similarity, the strain SN6^T^ exhibited 97.5% identity with *Vitreoscilla stercoraria* strain ATCC_15218 (L06174), the phylogenetically closest species with standing in nomenclature. The predominant fatty acid was hexadecenoic acid (31%). The genomic DNA G + C content of the strain SN6^T^ was 49.4 mol %. After analysis of taxonogenomic data, phenotypic and biochemical characteristics, we concluded that strain SN6^T^ represents a new species of the genus *Vitreoscilla* for which the name *Vitreoscilla massiliensis* sp.nov is proposed. The type strain is SN6^T^ (=CSUR P2036 = LN870312 = DSM 100958).

## Introduction

This strain was isolated from the stool specimen of an obese Amazonian patient as part of the culturomics study [1] to search for microaerophilic bacteria from human gut. The genus *Vitreoscilla* was first described by Pringsheim in 1951, after having proposed the family *Vitreoscillaceae* in 1949. In 1986, Strohl et al. proposed three new species with validated names (*Vitreoscilla stercoraria*, *Vitreoscilla beggiatoides* and *Vitreoscilla filiformis*) of this genus [2]. In 2013, through the use of new-generation sequencing tools, the genus *Vitreoscilla* was placed in the *Neisseriaceae* family on the basis of its branching in the 16S rRNA gene tree [3]. Within its clade, members of the genus were the only ones capable of evolving in different habitats.

Since the use of the culturomic concept, the repertoire of bacteria isolated from the human digestive microbiota [4] has expanded considerably. The characterization of these new species is based on a ribosomal RNA sequencing coupled with a taxonogenomics description, a strategy combining a comparison of genomic analysis and phenotypic characteristics, including the matrix-assisted laser desorption/ionization time-of-flight mass spectrometry (MALDI-TOF) spectrum. In the present study, we used this approach to facilitate the identification and the description of this novel species named *Vitreoscilla massiliensis* sp.nov.

## Materials and Methods

**Fig. 1:**
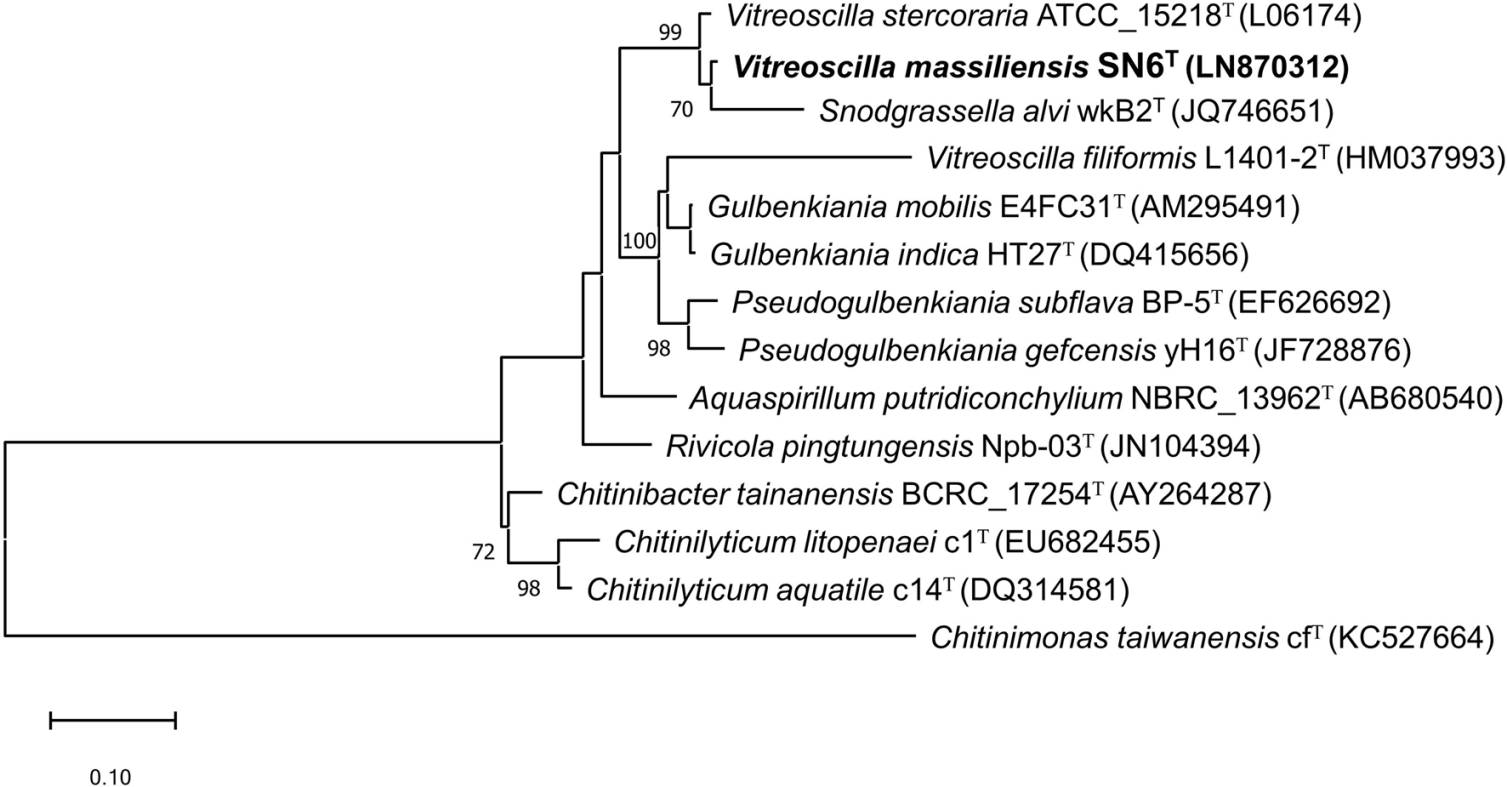
Phylogenetic tree showing the position of *Vitreoscilla massiliensis* SN6^T^ relative to other phylogenetically close neighbors. Sequences were aligned using CLUSTALW, and phylogenetic inferences are obtained with kimura two-parameter models using the maximum-likelihood method within the MEGA software. Numbers at nodes are percentages of bootstrap values obtained by repeating analysis 1,000 times to generate majority consensus tree. Scale bar indicates 1% nucleotide sequence divergence. The scale bar represents 500 nm

*Vitreoscilla massiliensis* SN6^T^ was isolated by cultivation on 5% sheep blood agar under microaerophilic conditions at 37 °C for 48 h and the strain could not be identified by Matrix-assisted laser desorption/ionization time-of-flight mass spectrometry.

(MALDI-TOF MS). The bacterial spectrum obtained was incremented in our database and its comparison with those of BioTyper database spectra and our own collection did not allow for its identification. Sequencing of 16S rRNA gene of the strain SN6^T^ showed a nucleotide sequence similarity of 97.5% with *V. stercoraria* strain (ATCC 15218) and *V. stercoraria* strain Göttingen 1488-6 (NR_025894.1), the phylogenetically closest species with standing in nomenclature (Fig. 1).

### Optimal Growth

Growth at various temperatures (28 °C, 37 °C, 42 °C, 45 °C) in different atmospheres (aerobic, microaerophilic using CampyGen from Thermo Scientific and anaerobic using AnaeroGenTM from bioMérieux) was tested by culture on Columbia agar (bioMérieux) after 48 h of incubation. The salinity acceptance limit of SN6^T^ strain was investigated by culture on a home-made culture medium consisting of a Columbia agar culture medium (Sigma-Aldrich, Saint-Quentin Fallavier, France) modified by adding (per liter) 5 g MgCl2 6H2O, 5 g MgSO4 7H2O, 2 g KCl, 1 g CaCl2 2H2O; 0.5 g NaBr, 0.5 g NaHCO3, and 2 g glucose with various NaCl concentrations 0, 5, 10, 25, 50, and 75 g/L. The pH range (6; 6.5; 7; 8.5) for growth was also determined and pH was adjusted by addition of HCl or NaOH.

### Biochemical and Chemotaxonomic Analysis

The abilities of the strain SN6^T^ to use various substrates as sole carbon sources were evaluated using the API 20NE and API 50CH (bioMérieux) and the presence of some enzyme activities using APIZYM following the manufacturer’s instructions. All tests were performed in duplicate. Susceptibility to antimicrobial agents was determined by the disk (i2a, Montpellier, France) diffusion method [5] on Mueller–Hinton agar in a Petri dish (BioMerieux) after 48 h of incubation at 37 °C under aerobic conditions. The interpretation of inhibition diameters to the manual measurement using a ruler was done using a Sirscan system© (i2a, Montpellier, France) according to the criteria proposed by the Comité de l’Antibiogramme of the French Society for Microbiology [6]. Cellular fatty acid methyl ester (FAME) analysis was performed by GC/MS. Two samples were prepared with approximately 65 mg of bacterial biomass per tube harvested from several culture plates. Fatty acid methyl esters were prepared as previously described by Sasser [7]. GC/MS analyses were carried out as previously described by Dione et al. [8]. Briefly, fatty acid methyl esters were separated using an Elite 5-MS column and monitored by mass spectrometry (Clarus 500—SQ 8 S, Perkin Elmer, Courtaboeuf, France). Spectral database search was performed using MS Search 2.0 operated with the Standard Reference Database 1A (NIST, Gaithersburg, USA) and the FAMEs mass spectral database (Wiley, Chichester, UK).

### Genome Sequencing and Assembly

DNA of strain SN6^T^ was extracted on the EZ1 biorobot (Qiagen) with a EZ1 DNA tissues kit after pretreatment by a lysozyme incubation at 37 °C, as previously described [9]. Genomic DNA (gDNA) was quantified by a Qubit assay with the high-sensitivity kit (Life technologies, Carlsbad, CA, USA) and sequenced on the MiSeq Technology (IlluminaInc, San Diego, CA, USA) with the mate pair strategy, as previously described [9]. The gDNA was barcoded in order to be mixed with 11 other projects with the Nextera Mate Pair sample prep kit (Illumina). The assembly of the genome was carried out with the help of a pipeline that allowed the creation of an assembly with different softwares (Velvet [10], Spades [11] and Soap Denovo [12], on trimmed (MiSeq and Trimmomatic [13] softwares) or untrimmed data (only MiSeq software). For each of the six assemblies performed, GapCloser [12] was used to reduce gaps. Then, contamination with Phage Phix was identified (blastn against Phage Phix174 DNA sequence) and eliminated. Finally, scaffolds whose size was less than 800 bp were removed and scaffolds whose depth value was lower than 25% of the mean depth were removed (identified as possible contaminants). The best assembly was selected using different criteria (number of scaffolds, N50 and number of N). Spades gave the best assembly of this strain, with a depth coverage of 98.

### Genome Annotation and Comparison

We used Prodigal as predicting tool of open reading frames (ORFs) [14] with default parameters. The predicted ORFs were excluded if they spanned a sequencing gap region (contained N). Using BLASTP, predicted bacterial protein sequences were blasted against GenBank and clusters of orthologous groups (COG) databases, DNA G + C content was identified by The RAST Server [15], and the tRNAs and rRNAs were predicted using the tRNAScan-SE [16] and RNAmmer tools [17], respectively. SignalP was used for Signal peptides prediction [18], the number of transmembrane helices was predicted using TMHMM [19], ORFans were identified if their BLASTP E-value was lower than 1e03 for alignment length greater than 80 amino acids. If alignment lengths were smaller than 80 amino acids, we used an *E*-value of 1e-05. Artemis [20] and DNA Plotter [21] were used for data management and visualization of genomic features, respectively. The Average Nucleotide identity at the genome level between *V. massiliensis* SN6 CZPV00000000.1, *V. stercoraria* ATCC_15218 (ARNN00000000.1), *V. filiformis* ATCC_43190 ( CP022423.1), *Gulbenkiania mobilis* E4FC31 (LIVN00000000.1), *Chitinilyticum litopenaei* DSM_21440 (ATZJ00000000.1), *Chitinilyticum aquatile* c14 (AUMS00000000.1), *Chitinibacter tainanensis* BCRC_17254 (AUCN00000000.1), and *Snodgrassella alvi* wkB2_wkB2 (CP007446.1) was estimated using Orthologous Average nucleotide identity tool (OAT) [22].

## Results and Discussion

Based on the sequence similarity threshold values of the 16S rRNA gene that delineate a new species according to the recommendations of Stackebrandt and Ebers [23], the strain SN6^T^ can, therefore, be classified as a new species of the genus *Vitreoscilla* and was accordingly named *V. massiliensis* SN6^T^ [24].

### Biochemical and Chemotaxonomic Analyses

API ZYM tests show positive reactions for esterase, esterase lipase, leucine arylamidase, acid phophatase, and naphthol-AS-BI-phosphohydrolase. In API 50CH, no substrate fermentation was observed and in API 20NE assimilation of substrates was observed for L-arginine dihydrolase and potassium gluconate. Some phenotypic characteristics of SN6^T^ with those of closely related species are presented in Table 1. The most abundant fatty acid is hexadecenoic acid (31%). Several hydroxyl fatty acids such as C_12:0_ 3-OH (4.5 ± 1.0) and C_14:0_ 3-OH (2.9 ± 0.1) are described. Other fatty acids such as 9-Hexadecenoic acid (22.0 ± 0.5), Dodecanoic acid (10.2 ± 0.6), 2-hexyl-cyclopropaneoctanoic acid (8.8 ± 0.4), Octadecenoic acid (8.0 ± 0.2), Pentadecanoic acid (5.5 ± 0.2), Tetradecanoic acid (3.3 ± 0.3), and Heptadecanoic acid (1.1 ± 0.1) were detected. The strain SN6^T^ was resistant to Oxacillin and Metronidazole, but susceptible to other antibiotics tested.

**Table 1:** Differential characteristics of *Vitreoscilla massiliensis* SN6^T^*, Vitreoscilla stercoraria* ATCC 15218, *Vitreoscilla filiformis* ATCC 15551, *Vitreoscilla beggiatoides* B23SS, *Gulbenkiania mobilis* E4FC31, *Chitinibacter tainanensis* BCRC 17254, *Chitinilyticum litopenaei* DSM _21440_c1, and *Snodgrassella alvi* wkB2 wkB2

Properties	*V.massiliensis*	*V. stercoraria*^a^	*V.filiformis*^b^	*V.beggiatoides*^c^	*G. mobilis*^d^	*C.litopenaei*^e^	*C.tainanensis*^f^	*S. alvi*^g^
Cell diameter (µm)	0.5	1.0	1.0–1.5	2.5–3	0.2–0.4	0.3–0.5	0.5–0.9	0.4
Oxygen requirement	Aerobic/Microaerophilic	Aerobic	Aerobic/Microaerophilic	Aerobic/Microaerophilic	Aerobic	Aerobic/Anaerobic	Aerobic	Microarophilic
Motility	–	+	+	+	+	+	+	-
Endospore formation	–	Na	Na	Na	–	–	Na	Na
pH	7–7.5	7.5–7.7	7.5	7.5	5.5–9.0	7–11	5.5–9	6.0–6.5
NaCl % (*w*/*v*)	0–0.5	Na	0	0.5	1.0	0–0.75	Na	Na
Indole	–	Na	Na	Na	–	–	–	–
Production of Alkaline phosphatase	–	Na	Na	Na	Na	+	Na	Na
Catalase	–	–	–	–	+	+	+	+
Oxidase	–	–	+	+	+	–	+	–
Nitrate reductase	–	–	+	+	–	+	–	+
Urease	–	Na	Na	Na	–	–	–	+
β-galactosidase	–	Na	Na	Na		–	–	–
N-acetyl-glucosamine	–	Na	Na	Na	–	+	+	Na
Acid from L-Arabinose	+	–	–	–	–	–	–	–
Trehalose	–	–	–	–	–	+	–	Na
D-mannose	+	–	–	–	–	+	–	–
Mannitol	+	–	–	–	–	–	–	–
D-glucose	+	–	+	–	–	+	–	–
D-fructose	–	–	–	–	–	+	–	–
Maltose	+	–	–	–	–	+	–	–
D-lactose	–	–	–	–	–	–	Na	Na
D-raffinose	–	Na	–	–	–	–	Na	Na
Habitat	Human gut	Dung of Cow	Freshwater sediments	Sandy sediments	Wastewater	Freshwater pond	Soil	Gut of Bees

*Na* not available

^a^Data from Mayfield et al. [25]

^b, c^Data from Strohl et al. [2]

^d^Data from Vaz-Moreira et al. [26]

^e^Data from Chang et al. [27]

^f^Data from Chern et al. [28]

^g^Data from Kwong et al. [29]

### Genome Properties

**Fig. 2:**
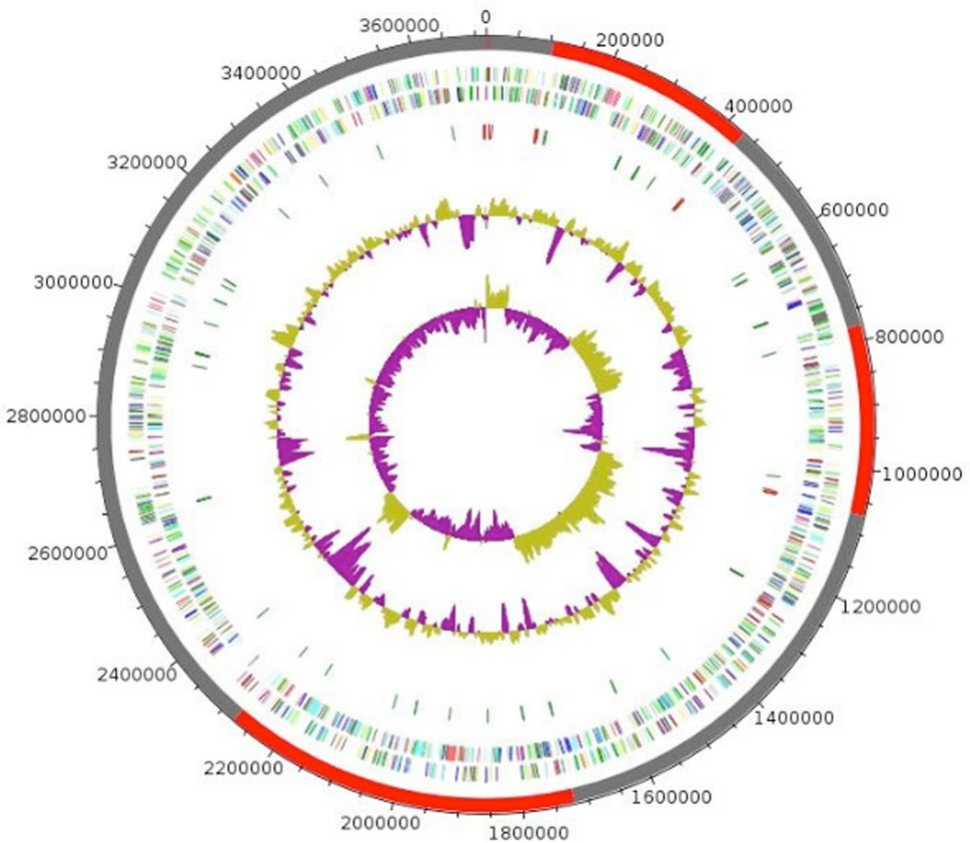
Graphical circular map of the genome of *Vitreoscilla massiliensis* strain SN6^T^ from outside to the center: genes on the forward strand colored by COG categories (only genes assigned to COG), genes on the reverse strand colored by COG categories (only gene assigned to COG), RNA genes (tRNAs green, rRNAs red), GC content, and GC skew (Color figure online)

The genome is 3,716,289 bp long with 49.4% GC content (Fig. 2, Table 2). It is composed of 10 scaffolds (composed of 13 contigs). Of the 3 716 predicted genes, 3 627 were protein-coding genes and 89 were RNAs (5 genes are 5S rRNA, 5 genes are 16S rRNA, 5 genes are 23S rRNA and 74 genes are TRNA genes). A total of 2,263 genes (62.3%) were assigned with putative function (by cogs or by NR blast). 475 genes were identified as ORFans (13.1%). The remaining genes were described as hypothetical proteins (744 genes ≥ 20.5%). A summary of the distribution of *V. massiliensis* genes into the different COGs categories is presented in Table 3.

**Table 2:** Nucleotide content and gene count levels of the genome

Attribute	Genome (total)	
	Value	% of total^a^
Size (bp)	3,716,289	100
G+C content (%)	1,836,063	49.42
Coding region (bp)	3,249,937	87.45
Total genes	3,716	100
RNA genes	89	2.39
Protein-coding genes	3,627	100
Genes with function prediction	2,263	62.39
Genes assigned to COGs	2,184	60.21
Genes with peptide signals	677	18.66
Genes with transmembrane helices	774	21.33
Genes associated to virulence	715	19.71
ORFn genes	475	13.09
Genes associated with PKS or NRPS	20	0.55
Genes associated to toxine/antitoxine	115	3.17

^a^The total is based on either the size of the genome in base pairs or the total number of protein-coding genes in the annotated genome

**Table 3:** Number of genes associated with the 25 general COG functional categories

Code	Value	% of total	Description
[J]	213	5.872622	Translation
[A]	1	0.027570996	Rna processing and modification
[K]	121	3.3360906	Transcription
[L]	88	2.4262476	Replication, recombination and repair
[B]	4	0.110283986	Chromatin structure and dynamics
[D]	32	0.8822719	Cell cycle control, mitosis and meiosis
[Y]	0	0	Nuclear structure
[V]	44	1.2131238	Defense mechanisms
[T]	85	2.3435347	Signal transduction mechanisms
[M]	140	3.8599393	Cell wall/membrane biogenesis
[N]	29	0.7995589	Cell motility
[Z]	0	0	Cytoskeleton
[W]	19	0.52384895	Extracellular structures
[U]	27	0.7444169	Intracellular trafficking and secretion
[O]	97	2.6743865	Post-translational modification, protein turnover, chaperones
[X]	77	2.1229665	Mobilome, prophages, transposons
[C]	177	4.8800664	Energy production and conversion
[G]	125	3.4463744	Carbohydrate transport and metabolism
[E]	287	7.9128757	Amino acid transport and metabolism
[F]	64	1.7645438	Nucleotide transport and metabolism
[H]	119	3.2809484	Coenzyme transport and metabolism
[I]	133	3.6669421	Lipid transport and metabolism
[P]	136	3.7496552	Inorganic ion transport and metabolism
[Q]	90	2.4813895	Secondary metabolites biosynthesis, transport and catabolism
[R]	234	6.4516125	General function prediction only
[S]	145	3.9977942	Function unknown
_	1443	39.784946	Not in COGs

### Genome Comparison

**Fig. 3:**
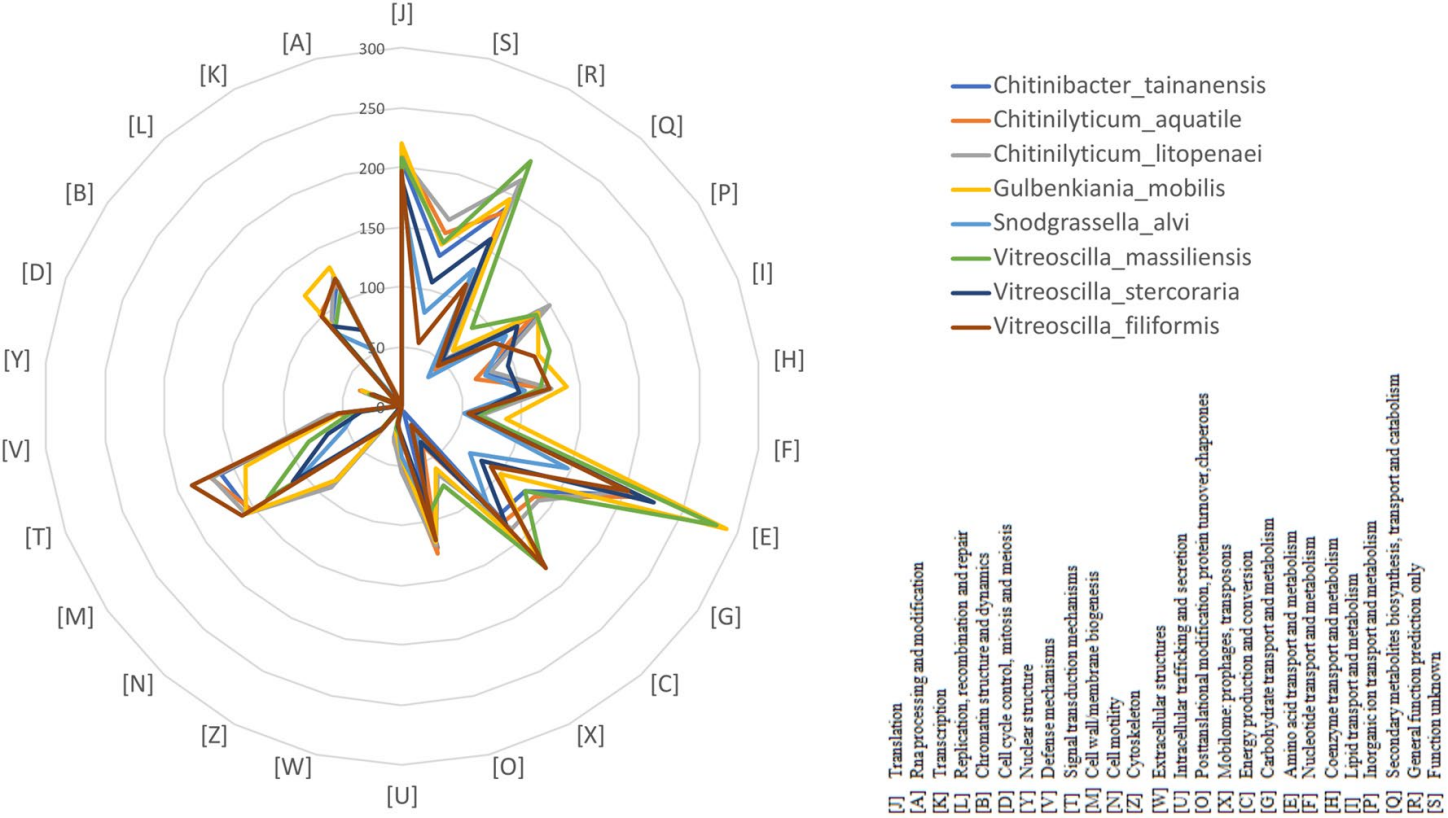
Distribution of functional classes of predicted genes according to the clusters of orthologous groups of proteins

**Fig.4:**
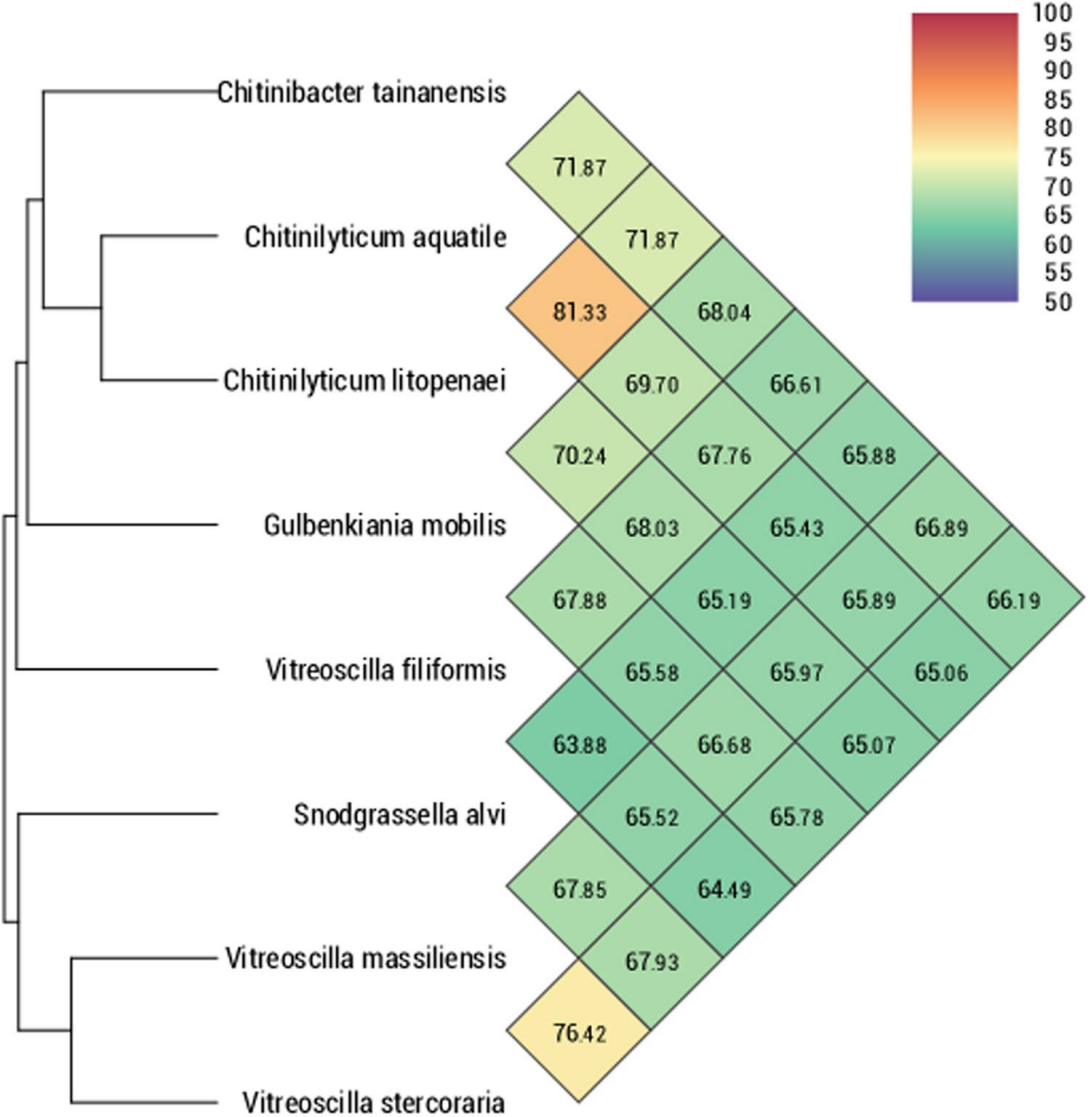
Heatmap generated with OrthoANI values calculated using the OAT software between *Vitreoscilla* species and other closely related species with standing in nomenclature

The draft genome sequence and the G + C content of *V. massiliensis* (3.71 MB and 49.4%) is larger than that of *V. stercoraria* (2.58 MB and 43.9% respectively)*.* Also, the gene content of *V. massiliensis* is larger than that of (3,627and 2,440 respectively)*.* The distribution of genes into COG categories was similar in all 7 compared genomes (Fig. 3). All genomes were compared with *V. massiliensis* using Orthologous average nucleotide identity. The OrthoANI analysis showed that identity nucleotide value is 76.4% with *V. stercoraria* which is lower than 95% (Fig. 4). Likewise, we obtained similar results for the analysis of the digital DNA-DNA hybridization (dDDH) with 31.60% between *V. massiliensis* and *V. stercoraria* (Table 4).

**Table 4:** Pairwise comparison of *Vitreoscilla massiliensis* SN6^T^ with other species using GGDC, formula 2(DNA-DNA hybridization estimates based on identities/HSP length)

	*C.tainanensis*	*C.aquatile*	*G.mobilis*	*S.alvi*	*C.litopenaei*	*V.stercoraria*	*V.filiformis*
*V.massiliensis*	23.20% 2.4±	30.00% 2.45±	29.40% 2.45±	26.80% 2.45±	30.80% 2.45±	21.50% 2.35±	28.30% 2.45±
*C.tainanensis*		18.80% 2.3±	19.30% 2.25±	31.10% 2.45±	19.20% 2.3±	19.70% 2.3±	26.30% 2.4±
*C.aquatile*			18.30% 2.25±	37.20% 2.5 ±	25.60% 2.45±	28.40% 2.45±	19.50% 2.3±
*G.mobilis*				29.30% 2.45±	18.7% 2.25±	31.20% 2.45±	18.50% 2.3±
*S.alvi*					33.8% 2.5±	24.00% 2.35±	29.60% 2.45±
*C.litopenaei*						32.9% 2.5±	18.40% 2.25±
*V.stercoraria*							31.60% 2.45±

*V.massiliensis* *Vitreoscilla massiliensis* SN6^T^, *G.mobilis* *Gulbenkiania mobilis* E4FC31, *C.litopenaei* *Chitinilyticum litopenaei* DSM _21440_c1, *S.alvi* *Snodgrassella alvi* wkB2 wkB2, *C.tainanensis* *Chitinibacter tainanensis* BCRC 17254, *C.aquatile* *Chitinilyticum aquatile* c14, *V.stercoraria* *Vitreoscilla stercoraria* ATCC 15218 and *V*.*filiformis* *Vitreoscilla filiformis* ATCC 15551

## Conclusion

Based on the phenotypic characteristics, and phylogenetic and genomic analyses of strain SN6^T^, we suggest the creation of a new species within the *Vitreoscilla genus*, for which the name *V. massiliensis* sp. nov., is proposed.

## Description of *Vitreoscilla massiliensis* sp. nov.

*Vitreoscilla massiliensis* (mas.si.li.en’sis. L. fem. adj. *massiliensis*, of Massilia, the Latin name of Marseille where strain SN6^T^ was first isolated).

Cells are Gram-negative (0.5 × 1.5–2 µm), non-motile, non-spore-forming , and often occur in a long chain under electron microscopy. *V. massiliensis* SN6^T^ grows at 28–37 °C and pH 7–7.5 and does not grow above 0.5% salinity. *V. massiliensis* SN6^T^ grows under microaerophilic atmosphere and a lower growth was observed under anerobic conditions. On agar plates, colonies were gray, smooth, and hemolytic with 0.5 to 1 mm in diameter after 48 h of incubation under aerobic conditions. They are catalase and oxidase negative. Tests were negative for urease, the reduction of nitrates, indole production, and fermentation of β-galactosidase. API 50CH shows that the carbohydrates provided by this panel were not used. *V. massiliensis* SN6^T^ is susceptible to Vancomycin (0.5), Cefotaxime (0.94), Tobamycin (0.38 µg), Fosfomycin (16 µg), Teicoplanin (1.5 µg), Rifampicin (0.29 µg), Colistin (0.32 µg), Imipenem (0.23 µg), Erythromycin (0.25 µg), Ceftriaxone (0.32 µg), and resistant to Oxacillin and Metronidazole. Major fatty acids are hexadecanoic acid (C_16:00_), 9-hexadecenoic acid (C_16:1n7_), and an unusual cylo fatty acid named 2-hexyl-cyclopropaneoctanoic acid (C_16:0_ 9,10-methylene). The DNA G + C content is about 49.4%.

The type strain is SN6^T^ (=CSUR P2036 = LN870312 = DSM 100958) and was isolated from the stool specimen of an obese Amazonian patient.

## Abbreviations

CSUR: Collection de Souches de l’Unité des Rickettsies
DSM: Deutsche Sammlung von Mikroorganismen
FAME: Fatty acid methyl ester
GC/SM: Gaz chromatography/mass spectrometry
OrthoANI: Orthologous average nucleotide identity
COGs: Clusters of orthologous groups
NR BLAST: Non-redundant protein sequence basic local alignment search tool
ORF: Open reading frame
GGDC: Genome-to-genome distance calculator
